# 1-Cyclo­propyl-6-fluoro-7-(4-nitro­so­piperazin-1-yl)-4-oxo-1,4-dihydro­quinoline-3-carboxylic acid

**DOI:** 10.1107/S1600536809029729

**Published:** 2009-07-31

**Authors:** Tao Li, Li-Rong Tang, Qiao-Ling Zeng, Wei-Jin Chen, Biao Huang

**Affiliations:** a School of Life Sciences, Fujian Agriculture and Forestry University, Fuzhou, Fujian 350002, People’s Republic of China; b Material Engineering College, Fujian Agriculture and Forestry University, Fuzhou, Fujian 350002, People’s Republic of China

## Abstract

The title compound, C_17_H_17_FN_4_O_4_, is a derivative of ciprofloxacin [1-cyclo­propyl-6-fluoro-4-oxo-7-(1-piperazin­yl)-1,4-dihydro­quinoline-3-carboxylic acid]. The crystal packing is stabilized by inter­molecular C—H⋯O hydrogen bonds together with π–π electron ring inter­actions [centroid–centroid separations between quinoline rings of 3.5864 (11) and 3.9339 (13) Å]. A strong intra­molecular O—H⋯O hydrogen bonds is present as well as an intra­molecular C—H⋯F inter­action.

## Related literature

For the biological activity of ciprofloxacin compounds, see: Neu (1987 ▶). For related structures, see: Turel *et al.* (1996 ▶); Drevenšek *et al.* (2003 ▶); Li *et al.* (2005 ▶); Lou *et al.* (2007 ▶). The nitroso-group geometry is similar to that observed in 1,4-dinitro­sopiperazine, see: Sekido *et al.* (1985 ▶).
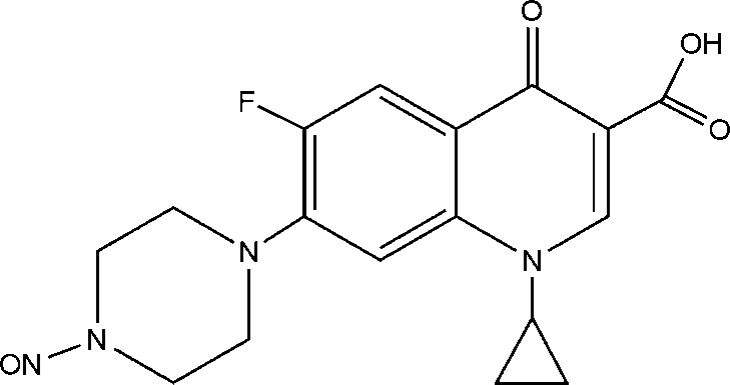


## Experimental

### 

#### Crystal data

C_17_H_17_FN_4_O_4_
*M* *_r_* = 360.35
Triclinic, 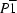

*a* = 8.378 (3) Å
*b* = 9.625 (4) Å
*c* = 10.328 (4) Å
α = 102.99 (2)°
β = 96.089 (14)°
γ = 97.392 (16)°
*V* = 797.0 (6) Å^3^
*Z* = 2
Mo *K*α radiation
μ = 0.12 mm^−1^
*T* = 293 K
0.2 × 0.2 × 0.2 mm

#### Data collection

Rigaku Mercury CCD/AFC diffractometer
Absorption correction: multi-scan (*CrystalClear*; Rigaku, 2007 ▶) *T*_min_ = 0.976, *T*_max_ = 0.977
6267 measured reflections
3631 independent reflections
2568 reflections with *I* > 2σ(*I*)
*R*_int_ = 0.031

#### Refinement

*R*[*F* ^2^ > 2σ(*F* ^2^)] = 0.067
*wR*(*F* ^2^) = 0.207
*S* = 1.06
3631 reflections
236 parameters
H-atom parameters constrained
Δρ_max_ = 0.52 e Å^−3^
Δρ_min_ = −0.35 e Å^−3^

### 

Data collection: *CrystalClear* (Rigaku, 2007 ▶); cell refinement: *CrystalClear*; data reduction: *CrystalClear*; program(s) used to solve structure: *SHELXS97* (Sheldrick, 2008 ▶); program(s) used to refine structure: *SHELXL97* (Sheldrick, 2008 ▶); molecular graphics: *SHELXL97* and *DIAMOND* (Brandenburg, 2005 ▶); software used to prepare material for publication: *SHELXTL* (Sheldrick, 2008 ▶).

## Supplementary Material

Crystal structure: contains datablocks global, I. DOI: 10.1107/S1600536809029729/fb2160sup1.cif

Structure factors: contains datablocks I. DOI: 10.1107/S1600536809029729/fb2160Isup2.hkl

Additional supplementary materials:  crystallographic information; 3D view; checkCIF report

## Figures and Tables

**Table 1:** Hydrogen-bond geometry (Å, °)

*D*—H⋯*A*	*D*—H	H⋯*A*	*D*⋯*A*	*D*—H⋯*A*
O2—H18⋯O3	0.84	1.78	2.562 (2)	153
C15—H15*A*⋯O2^i^	0.99	2.50	3.405 (3)	151
C15—H15*B*⋯O3^ii^	0.99	2.51	3.385 (3)	147
C16—H16*A*⋯O1^iii^	0.99	2.60	3.264 (3)	125
C16—H16*B*⋯F1	0.99	2.14	2.852 (3)	128

Symmetry codes: (i) 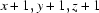; (ii) 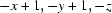; (iii) 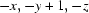.

**Fig. 1.**
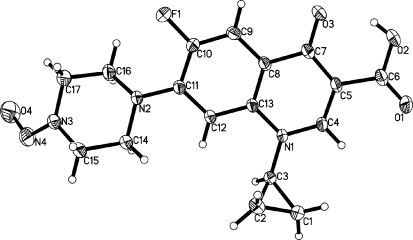
View of the title molecule. The displacement ellipsoids are drawn at the 30% propability level

**Fig. 2.**
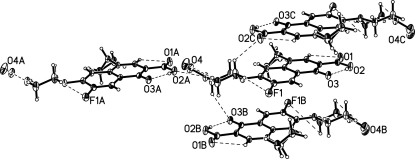
The intramolecular and intermoleclar hydrogen bonds or interactions (the dashed lines) in title compound (see Table 1). Symmetry codes: (A) x+1, y+1, z+1; (B) x+1, y+1, z; (C) x, y+1, z

**Fig. 3.**
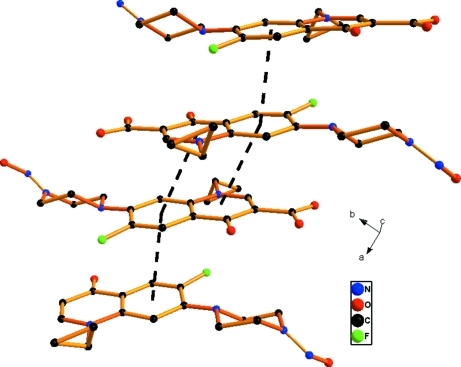
The packing of title molecules, showing the π-π-electron ring interactions. The H atoms have been omitted for clarity

## supplementary crystallographic information

### Comment

Ciprofloxacin (1-cyclopropyl-6-fluoro -1,4-dihydro-4-oxo-7-(1-piperazinyl)-3-quinoline carboxylic acid) is used as an antibacterial agent. Ciprofloxacin is widely used in clinical practice for the treatment of certain diseases caused by some Gram negative and as well as Gram positive microorganisms (Neu, 1987). Recently, several structures containing ciprofloxacin have been reported (Turel *et al.*, 1996; Drevenšek *et al.*, 2003; Lou *et al.*, 2007).

Nitrosation of amines by nitrites takes place in acid medium. The nature of the product depends on the nature of the initial amine. Commonly the secondary alkyl amines yield *N*-nitrosoamines. In our case, the *N*-nitrosation of ciprofloxacin occurs by ytterbium nitrate in nitric acid and results in the formation of ON-ciprofloxacin under hydrothermal reaction.

The title compound is composed of an essentially planar quinoline ring system [the mean deviation from best plane is 0.0274 (2) Å] which is substituted with cyclopropyl, fluoro, oxo, carboxyl and nitrosopiperazinium groups (Fig. 1). The bond distances and angles are in agreement with those in 1-cyclopropyl-6-fluoro-7- (4-formylpiperazin-1-yl)-4-oxo-1,4-dihydroquinoline-3-carboxylic acid (Li *et al.*, 2005).

In the title structure, the six-membered piperazinyl ring adopts a chair conformation. The nitroso-group geometry with the NO distance equal to 1.2382 (31) Å and O—N—N bond angle of 115.583 (20)° is similar to that observed in 1,4-dinitrosopiperazine (Sekido *et al.*, 1985).

For the hydrogen bonding, please see Tab. 1 that comprises intramolecular and intermolecular hydrogen bonds in the structure (Fig. 2). As shown in Fig. 3, the crystal packing is stabilized by π-π stacking interactions of the quinoline rings, in which the N1 ring (N1/C4—C6/C7—C13) stacks with the inversion-related N1 rings, with the centroid-centroid separations of 3.5864 (11) and 3.9339 (13) Å.

### Experimental

The title compound was hydrothermally synthesized under autogenous pressure. A mixture of C_17_H_18_FN_3_O_3_.HCl (ciprofloxacin hydrochloride) (50 mg, 0.14 mmol), Yb(NO_3_)_3_ (72 mg, 0.2 mmol), HNO_3_ (1 ml of 0.5 M), C_2_H_5_OH (4 ml) and H_2_O (8 ml) was sealed in a stainless reactor with a Teflon liner. The mixture was heated to 393 K for one day. After cooling at a rate of 10 K h^-1^ to room temperature, yellow needle crystals (average 4 mm long by 0.6 mm diameter) were separated by filtration, washed with distilled water and finally dried in air. Yield 75%, Anal. calc. for C_17_H_17_FN_4_O_4_: C, 56.66; H, 4.76; N, 15.55%; Found: C, 56.82; H, 4.75; N, 15.63%. IR (KBr pellet): 1719(*s*), 1627(*s*), 1489(*m*), 1454(*m*), 1334(*m*), 1339(*m*), 1257(*s*), 1152(*m*), 994(*m*), 896(*m*), 798(*m*), 743(*m*).

### Refinement

All the hydrogen atoms were discernible in the difference electron density maps. However, the hydrogens were situated into idealized positions and constrained by the riding model approximation: O—H_carboxyl_ = 0.84 [the command AFIX 147 of SHELXL-97 has been applied (Sheldrick, 2008)], C_aryl_—H_aryl_ = 0.95, C_methylene_—H_methylene_ = 0.99 and C_methine_—H_methine_ = 1.00 Å; *U*_iso_H = 1.2*U*_eq_(C); *U*_iso_H_carboxyl_ = 1.5*U*_eq_(O). The highest electron-density peak is situated 1.12 Å from H16A and the deepest hole 0.54 Å from C17.

### Crystal data

**Table:**

C_17_H_17_FN_4_O_4_	*Z* = 2
*M**_r_* = 360.35	*F*(000) = 376
Triclinic, *P*1	*D*_x_ = 1.502 Mg m^−^^3^
Hall symbol: -P 1	Mo *K*α radiation, λ = 0.71073 Å
*a* = 8.378 (3) Å	Cell parameters from 771 reflections
*b* = 9.625 (4) Å	θ = 2.0–27.4°
*c* = 10.328 (4) Å	µ = 0.12 mm^−^^1^
α = 102.99 (2)°	*T* = 293 K
β = 96.089 (14)°	Block, yellow
γ = 97.392 (16)°	0.2 × 0.2 × 0.2 mm
*V* = 797.0 (6) Å^3^	

### Data collection

**Table:**

Rigaku Mercury CCD/AFC diffractometer	3631 independent reflections
Radiation source: fine-focus sealed tube	2568 reflections with *I* > 2σ(*I*)
graphite	*R*_int_ = 0.031
φ and ω scans	θ_max_ = 27.4°, θ_min_ = 2.5°
Absorption correction: multi-scan (*CrystalClear*; Rigaku, 2007)	*h* = −10→10
*T*_min_ = 0.976, *T*_max_ = 0.977	*k* = −12→12
6267 measured reflections	*l* = −13→11

### Refinement

**Table:**

Refinement on *F*^2^	Primary atom site location: structure-invariant direct methods
Least-squares matrix: full	Secondary atom site location: difference Fourier map
*R*[*F*^2^ > 2σ(*F*^2^)] = 0.067	Hydrogen site location: difference Fourier map
*wR*(*F*^2^) = 0.207	H-atom parameters constrained
*S* = 1.06	*w* = 1/[σ^2^(*F*_o_^2^) + (0.1165*P*)^2^ + 0.047*P*] where *P* = (*F*_o_^2^ + 2*F*_c_^2^)/3
3631 reflections	(Δ/σ)_max_ < 0.001
236 parameters	Δρ_max_ = 0.52 e Å^−^^3^
0 restraints	Δρ_min_ = −0.35 e Å^−^^3^
67 constraints	

### Special details

**Table:**

Geometry. All e.s.d.'s (except the e.s.d. in the dihedral angle between two l.s. planes) are estimated using the full covariance matrix. The cell e.s.d.'s are taken into account individually in the estimation of e.s.d.'s in distances, angles and torsion angles; correlations between e.s.d.'s in cell parameters are only used when they are defined by crystal symmetry. An approximate (isotropic) treatment of cell e.s.d.'s is used for estimating e.s.d.'s involving l.s. planes.
Refinement. Refinement of *F*^2^ against ALL reflections. The weighted *R*-factor *wR* and goodness of fit *S* are based on *F*^2^, conventional *R*-factors *R* are based on *F*, with *F* set to zero for negative *F*^2^. The threshold expression of *F*^2^ > σ(*F*^2^) is used only for calculating *R*-factors(gt) *etc*. and is not relevant to the choice of reflections for refinement. *R*-factors based on *F*^2^ are statistically about twice as large as those based on *F*, and *R*- factors based on ALL data will be even larger.

### Fractional atomic coordinates and isotropic or equivalent isotropic displacement parameters (Å^2^)

**Table:**

	*x*	*y*	*z*	*U*_iso_*/*U*_eq_	
N1	0.14474 (19)	0.38921 (17)	0.16119 (15)	0.0330 (4)	
O2	−0.1340 (2)	0.09746 (18)	−0.25939 (15)	0.0561 (5)	
H18	−0.0827	0.1674	−0.2814	0.084*	
O3	0.0676 (2)	0.32428 (17)	−0.25086 (13)	0.0472 (4)	
C8	0.2117 (2)	0.4611 (2)	−0.03990 (18)	0.0331 (4)	
F1	0.51960 (16)	0.74426 (14)	−0.07933 (12)	0.0505 (4)	
C13	0.2335 (2)	0.48548 (19)	0.10118 (18)	0.0318 (4)	
C11	0.4372 (2)	0.6987 (2)	0.1245 (2)	0.0342 (4)	
C5	0.0120 (2)	0.2477 (2)	−0.05450 (19)	0.0356 (5)	
O1	−0.1742 (2)	0.03235 (18)	−0.07173 (17)	0.0580 (5)	
C12	0.3415 (2)	0.6063 (2)	0.18211 (19)	0.0344 (4)	
H12A	0.3488	0.6247	0.2770	0.041*	
C9	0.3081 (3)	0.5551 (2)	−0.0978 (2)	0.0372 (5)	
H9A	0.2962	0.5415	−0.1925	0.045*	
N2	0.5528 (2)	0.81427 (18)	0.20147 (16)	0.0380 (4)	
C7	0.0944 (2)	0.3414 (2)	−0.1242 (2)	0.0361 (5)	
C10	0.4184 (3)	0.6654 (2)	−0.0179 (2)	0.0361 (5)	
C4	0.0408 (2)	0.2758 (2)	0.0830 (2)	0.0359 (5)	
H4A	−0.0163	0.2111	0.1257	0.043*	
C6	−0.1066 (3)	0.1168 (2)	−0.1269 (2)	0.0428 (5)	
N3	0.7368 (3)	1.0685 (2)	0.37104 (18)	0.0492 (5)	
C3	0.1612 (3)	0.4155 (2)	0.30774 (19)	0.0373 (5)	
H3A	0.1132	0.4997	0.3544	0.045*	
C14	0.5794 (3)	0.8301 (2)	0.3473 (2)	0.0473 (6)	
H14A	0.5869	0.7346	0.3663	0.057*	
H14B	0.4859	0.8666	0.3870	0.057*	
C2	0.3112 (3)	0.3878 (3)	0.3816 (2)	0.0512 (6)	
H2A	0.3549	0.4545	0.4696	0.061*	
H2B	0.3943	0.3493	0.3283	0.061*	
C15	0.7354 (3)	0.9344 (2)	0.4114 (2)	0.0487 (6)	
H15A	0.7434	0.9535	0.5103	0.058*	
H15B	0.8306	0.8901	0.3840	0.058*	
C16	0.5548 (3)	0.9558 (3)	0.1687 (2)	0.0570 (7)	
H16A	0.4633	1.0008	0.2039	0.068*	
H16B	0.5390	0.9417	0.0700	0.068*	
C17	0.7110 (3)	1.0550 (3)	0.2268 (2)	0.0574 (7)	
H17A	0.8022	1.0156	0.1856	0.069*	
H17B	0.7058	1.1509	0.2082	0.069*	
C1	0.1525 (3)	0.2910 (3)	0.3709 (2)	0.0551 (7)	
H1A	0.1379	0.1929	0.3111	0.066*	
H1B	0.0986	0.2981	0.4524	0.066*	
N4	0.7544 (3)	1.1903 (2)	0.4635 (2)	0.0654 (7)	
O4	0.7463 (3)	1.3001 (2)	0.4211 (2)	0.0895 (7)	

### Atomic displacement parameters (Å^2^)

**Table:**

	*U*^11^	*U*^22^	*U*^33^	*U*^12^	*U*^13^	*U*^23^
N1	0.0369 (9)	0.0264 (8)	0.0303 (8)	−0.0042 (6)	−0.0015 (7)	0.0040 (6)
O2	0.0664 (11)	0.0464 (10)	0.0397 (9)	−0.0092 (8)	−0.0087 (8)	−0.0047 (7)
O3	0.0588 (10)	0.0433 (9)	0.0306 (8)	−0.0015 (7)	−0.0052 (7)	0.0012 (6)
C8	0.0372 (10)	0.0277 (9)	0.0302 (10)	0.0036 (8)	−0.0017 (8)	0.0023 (7)
F1	0.0587 (8)	0.0475 (8)	0.0438 (7)	−0.0084 (6)	0.0111 (6)	0.0155 (6)
C13	0.0359 (10)	0.0249 (9)	0.0310 (10)	0.0015 (8)	0.0008 (8)	0.0029 (7)
C11	0.0373 (10)	0.0278 (9)	0.0340 (10)	0.0004 (8)	−0.0015 (8)	0.0061 (8)
C5	0.0382 (10)	0.0268 (10)	0.0348 (11)	0.0003 (8)	−0.0037 (8)	−0.0008 (8)
O1	0.0590 (11)	0.0456 (10)	0.0569 (10)	−0.0192 (8)	−0.0103 (8)	0.0103 (8)
C12	0.0416 (11)	0.0270 (9)	0.0295 (10)	−0.0019 (8)	0.0003 (8)	0.0025 (7)
C9	0.0455 (11)	0.0354 (10)	0.0293 (10)	0.0065 (9)	0.0029 (8)	0.0057 (8)
N2	0.0446 (10)	0.0308 (9)	0.0329 (9)	−0.0071 (7)	−0.0044 (7)	0.0074 (7)
C7	0.0390 (11)	0.0306 (10)	0.0336 (10)	0.0058 (8)	−0.0010 (8)	−0.0002 (8)
C10	0.0395 (10)	0.0312 (10)	0.0375 (11)	0.0008 (8)	0.0039 (8)	0.0115 (8)
C4	0.0361 (10)	0.0284 (10)	0.0380 (11)	−0.0023 (8)	−0.0013 (8)	0.0043 (8)
C6	0.0426 (11)	0.0340 (11)	0.0436 (12)	0.0024 (9)	−0.0064 (9)	0.0002 (9)
N3	0.0662 (13)	0.0354 (10)	0.0359 (9)	−0.0088 (9)	−0.0016 (9)	0.0013 (7)
C3	0.0473 (12)	0.0311 (10)	0.0294 (10)	−0.0022 (8)	0.0031 (8)	0.0045 (8)
C14	0.0608 (14)	0.0394 (12)	0.0337 (11)	−0.0112 (10)	−0.0030 (10)	0.0075 (9)
C2	0.0527 (14)	0.0551 (14)	0.0393 (12)	−0.0017 (11)	−0.0041 (10)	0.0086 (10)
C15	0.0607 (15)	0.0401 (12)	0.0364 (11)	−0.0056 (10)	−0.0068 (10)	0.0051 (9)
C16	0.0679 (16)	0.0395 (13)	0.0546 (14)	−0.0137 (11)	−0.0191 (12)	0.0179 (11)
C17	0.0767 (18)	0.0424 (13)	0.0451 (13)	−0.0128 (12)	−0.0004 (12)	0.0104 (10)
C1	0.0709 (16)	0.0458 (13)	0.0420 (12)	−0.0121 (12)	−0.0059 (11)	0.0150 (10)
N4	0.0859 (17)	0.0430 (12)	0.0536 (13)	−0.0081 (11)	0.0084 (12)	−0.0059 (10)
O4	0.132 (2)	0.0383 (11)	0.0856 (16)	0.0046 (12)	0.0002 (14)	0.0002 (10)

### Geometric parameters (Å, °)

**Table:**

N1—C4	1.346 (2)	C4—H4A	0.9500
N1—C13	1.405 (2)	N3—N4	1.315 (3)
N1—C3	1.466 (2)	N3—C15	1.442 (3)
O2—C6	1.330 (3)	N3—C17	1.456 (3)
O2—H18	0.8400	C3—C1	1.485 (3)
O3—C7	1.273 (2)	C3—C2	1.485 (3)
C8—C9	1.410 (3)	C3—H3A	1.0000
C8—C13	1.411 (3)	C14—C15	1.528 (3)
C8—C7	1.458 (3)	C14—H14A	0.9900
F1—C10	1.365 (2)	C14—H14B	0.9900
C13—C12	1.414 (3)	C2—C1	1.501 (3)
C11—C12	1.394 (3)	C2—H2A	0.9900
C11—N2	1.404 (2)	C2—H2B	0.9900
C11—C10	1.420 (3)	C15—H15A	0.9900
C5—C4	1.373 (3)	C15—H15B	0.9900
C5—C7	1.431 (3)	C16—C17	1.496 (3)
C5—C6	1.493 (3)	C16—H16A	0.9900
O1—C6	1.210 (3)	C16—H16B	0.9900
C12—H12A	0.9500	C17—H17A	0.9900
C9—C10	1.361 (3)	C17—H17B	0.9900
C9—H9A	0.9500	C1—H1A	0.9900
N2—C14	1.469 (3)	C1—H1B	0.9900
N2—C16	1.474 (3)	N4—O4	1.238 (3)
			
C4—N1—C13	119.38 (16)	C1—C3—C2	60.70 (16)
C4—N1—C3	120.52 (15)	N1—C3—H3A	115.5
C13—N1—C3	120.07 (15)	C1—C3—H3A	115.5
C6—O2—H18	109.5	C2—C3—H3A	115.5
C9—C8—C13	118.03 (17)	N2—C14—C15	110.91 (17)
C9—C8—C7	120.60 (17)	N2—C14—H14A	109.5
C13—C8—C7	121.36 (18)	C15—C14—H14A	109.5
N1—C13—C8	119.20 (16)	N2—C14—H14B	109.5
N1—C13—C12	120.02 (17)	C15—C14—H14B	109.5
C8—C13—C12	120.77 (17)	H14A—C14—H14B	108.0
C12—C11—N2	122.48 (18)	C3—C2—C1	59.64 (15)
C12—C11—C10	116.46 (17)	C3—C2—H2A	117.8
N2—C11—C10	120.98 (17)	C1—C2—H2A	117.8
C4—C5—C7	120.37 (17)	C3—C2—H2B	117.8
C4—C5—C6	117.68 (18)	C1—C2—H2B	117.8
C7—C5—C6	121.95 (18)	H2A—C2—H2B	114.9
C11—C12—C13	120.90 (18)	N3—C15—C14	110.6 (2)
C11—C12—H12A	119.6	N3—C15—H15A	109.5
C13—C12—H12A	119.6	C14—C15—H15A	109.5
C10—C9—C8	119.97 (18)	N3—C15—H15B	109.5
C10—C9—H9A	120.0	C14—C15—H15B	109.5
C8—C9—H9A	120.0	H15A—C15—H15B	108.1
C11—N2—C14	117.59 (15)	N2—C16—C17	111.9 (2)
C11—N2—C16	117.81 (17)	N2—C16—H16A	109.2
C14—N2—C16	111.17 (17)	C17—C16—H16A	109.2
O3—C7—C5	123.16 (18)	N2—C16—H16B	109.2
O3—C7—C8	121.43 (18)	C17—C16—H16B	109.2
C5—C7—C8	115.41 (17)	H16A—C16—H16B	107.9
C9—C10—F1	117.51 (17)	N3—C17—C16	108.62 (19)
C9—C10—C11	123.63 (18)	N3—C17—H17A	110.0
F1—C10—C11	118.81 (17)	C16—C17—H17A	110.0
N1—C4—C5	124.20 (18)	N3—C17—H17B	110.0
N1—C4—H4A	117.9	C16—C17—H17B	110.0
C5—C4—H4A	117.9	H17A—C17—H17B	108.3
O1—C6—O2	121.06 (19)	C3—C1—C2	59.66 (15)
O1—C6—C5	123.8 (2)	C3—C1—H1A	117.8
O2—C6—C5	115.15 (19)	C2—C1—H1A	117.8
N4—N3—C15	119.31 (19)	C3—C1—H1B	117.8
N4—N3—C17	125.3 (2)	C2—C1—H1B	117.8
C15—N3—C17	115.35 (18)	H1A—C1—H1B	114.9
N1—C3—C1	119.44 (18)	O4—N4—N3	115.6 (2)
N1—C3—C2	119.07 (18)		

### Hydrogen-bond geometry (Å, °)

**Table:**

*D*—H···*A*	*D*—H	H···*A*	*D*···*A*	*D*—H···*A*
O2—H18···O3	0.84	1.78	2.562 (2)	153
C4—H4A···O1	0.95	2.48	2.812 (3)	101
C15—H15A···O2^i^	0.99	2.50	3.405 (3)	151
C15—H15B···O3^ii^	0.99	2.51	3.385 (3)	147
C16—H16A···O1^iii^	0.99	2.60	3.264 (3)	125
C16—H16B···F1	0.99	2.14	2.852 (3)	128
C17—H17B···O4	0.99	2.30	2.692 (3)	102

Symmetry codes: (i) *x*+1, *y*+1, *z*+1; (ii) −*x*+1, −*y*+1, −*z*; (iii) −*x*, −*y*+1, −*z*.

## References

[bb1] Brandenburg, K. (2005). *DIAMOND* Crystal Impact GbR, Bonn, Germany.

[bb2] Drevenšek, P., Leban, I., Turel, I., Giester, G. & Tillmanns, E. (2003). *Acta Cryst.* C**59**, m376–m378. 10.1107/s0108270103016330 12944650

[bb3] Li, X.-W., Zhi, F., Shen, J.-H. & Hu, Y.-Q. (2005). *Acta Cryst.* E**61**, o2235–o2236.

[bb4] Lou, B., Boström, D. & Velaga, S. P. (2007). *Acta Cryst.* C**63**, o731–o733. 10.1107/S0108270107053218 18057626

[bb5] Neu, H. C. (1987). *Am. J. Med.* **82**, 395-404. 3578329

[bb6] Rigaku (2007). *CrystalClear* Rigaku Corporation, Tokyo, Japan.

[bb7] Sekido, K., Okamoto, K. & Hirokawa, S. (1985). *Acta Cryst.* C**41**, 741–743.

[bb8] Sheldrick, G. M. (2008). *Acta Cryst.* A**64**, 112–122. 10.1107/S0108767307043930 18156677

[bb9] Turel, I., Leban, I., Zupancic, M., Bukovec, P. & Gruber, K. (1996). *Acta Cryst.* C**52**, 2443–2445.

